# Burning Feet

**Published:** 1866-12

**Authors:** 


### BURNING FEET.

If the soles are dry and hot at bed time, rub patiently into each one of them, with the hand, half a teaspoonful of sweet oil night after night, until the difficulty is removed.

Some persons always have cold feet on getting into bed ; a robust person may remedy this in time by dipping both feet at a time in cold water just deep enough to cover the toes; let them remain in until thirty are counted, wipe dry, hold to the fire, and jump into bed.

Feeble persons and invalids should pursue a different course. Put both feet in hot water half leg deep; add hot water from time to time for fifteen minutes, so that the water shall be hotter when they are taken out then when they are put in, then dip them in cold water as before, while you count ten, wipe warm, and get into bed.

As cold feet induces a number of diseases, aggravates others, and delays the cure of all, it is woith all the trouble one can take if thereby, even in the course of months, the delightful condition can be brought about wherein the feet are in such a natural and healthful state, that the mind is never attracted towards them unpleasantly.

